# Orthopedic Implant Removal in the Department of Orthopedics of a Tertiary Care Centre of Nepal: A Descriptive Cross-sectional Study

**DOI:** 10.31729/jnma.6171

**Published:** 2021-02-28

**Authors:** Ravi Bhandari, Pravakar Dawadi, Mohit Thapa Magar, Ritesh Sinha, Nirab Kayastha, Rajesh Pratap Shah, Bishnu Babu Thapa, Sushil Rana Magar

**Affiliations:** 1 Department of Orthopedics, Nepalese Army Institute of Health Sciences, Shree Birendra Hospital, Chhauni, Kathmandu, Nepal; 2 Nepalese Army Institute of Health Sciences, Bhandarkhal, Kathmandu

**Keywords:** *elective surgical procedures*, *implants*, *orthopedic*, *surgery*

## Abstract

**Introduction:**

Implant removal surgery is one of the common surgical procedures done in orthopedics. Studies report that a major portion of orthopedic surgeries carried out in different institutions comprises implant removal procedures. This can be challenging in limited manpower and infrastructure availability scenarios, like in developing countries like Nepal. This study aims to study the prevalence of orthopedic implant removal procedures carried out among overall surgical procedures in the orthopedic department of a tertiary care center in Nepal.

**Methods:**

A descriptive cross-sectional study was performed on the medical records of the department of orthopedics of a tertiary care center after approval from the institutional review committee. The data included records from the starting of 2018 to the end of 2019. Data related to the number of implant removal procedures, types of implants, indications, fracture sites, anesthesia use, gender and age distribution were studied. Statistical Package for Social Sciences version 20 was used to study descriptive data.

**Results:**

Out of 2557 orthopedic operations carried out in the study duration, 458 (17.91%) of implant removal procedures were done in the department. The most common age group was the young adult age group, 255 (55.68%). Medium-sized implants were the commonly removed ones, 337 (73.58%). Elective procedures were the most common indication, 369 (80.57%).

**Conclusions:**

Implant removal procedures cover a major fraction of overall orthopedic operations carried out by the department, most of which are elective procedures. In limited-resource settings, this can be challenging, and a proper evaluation with counseling could be done before implant removal surgery.

## INTRODUCTION

Surgical stabilization of the fracture is one of the commonest operations in orthopedics and most of them involve the application of some type of implant. Implants are generally removed after fracture union when the purpose of the implant has been fulfilled. Orthopedic implant removal procedures are among the most carried out procedures in the department of orthopedics.^1^

There are not any standard evidence-based guidelines referring to the criteria for performing the implant removal surgery. The cause may vary from elective and implant-associated complications. A significant chunk of the workforce is occupied in orthopedics in implant removal procedures alone, and most of them are elective surgery.^2,3^ This often creates a scarcity of skilled manpower for other major orthopedic surgeries in limited-resource settings.

This study aims to find the prevalence of implant removal surgeries among overall surgical procedures in the orthopedics department of Shree Birendra Hospital, Chhauni.

## METHODS

A descriptive cross-sectional study was conducted in the department of orthopedics of Shree Birendra Hospital, Chhauni, Kathmandu, Nepal. The study was done upon the retrospective data of hospital records of the year 2018 and 2019 after the approval from the Institutional Review Committee. The sample size was calculated as follows;


$$\begin{array}{ll} \text{n} & ={\text{Z}}^{\text{2}}\times (\text{p}\times \text{q)}/{\text{e}}^{\text{2}}\\ & ={\left(1.96\right)}^{2}\times 0.5\times (1-0.5)/{\left(0.05\right)}^{\text{2}}\\ & =384 \end{array}$$

Where,

Z = 1.96 for confidence interval at 95%
p = prevalence 50% for maximum sample size
q = 1-p
e = margin of error 5%

The minimum sample size was calculated to be 384. Adding 20% of the non-response rate accounting for missing and incomplete data estimated minimum sample size was 461. The data related to all the orthopedic operations performed by the department of orthopedics during the study duration were included. Records without incomplete information and missing data were excluded. We took 2557 data of all the orthopedic operations carried out by the department accordingly.

The information related to the number of implant removal surgery procedures, type of implants used, indications behind removing those implants and sites of fractures for which the implant removal surgery had been carried out were studied. The related data were systematically entered in Statistical Package for Social Sciences (SPSS) version 20, and descriptive analysis was performed.

## RESULTS

A total of 2557 orthopedic operations were done during the study duration, out of which 1323 were in 2018 and 1234 in 2019. There was a total of 458 (17.91%) implant removal procedures carried out in the study period (Table 1).

**Table 1:** Number of implant removal procedures carried out.

Year	n (%)
2018 A.D.	239 (52.18)
2019 A.D.	219 (47.82)
Total	458 (100)

Most of the implant removal procedures were done among the age group of 17-39 years 255 (55.68%) and the least among the people of age ≥60 years 32 (6.99%) (Table 2).

**Table 2:** Age distribution of patients whose implants were removed.

Age group	n (%)
≤16 years (Pediatric age group)	51 (11.14)
17-39 years (Young adult)	255 (55.68)
40-59 years (Middle-aged)	120 (26.20)
≥60 years (Elderly)	32 (6.99)
Total	458 (100)

There were 351 (76.64%) males out of those patients undergoing elective procedures (Table 3).

**Table 3:** Gender distribution among patients whose implants were removed.

Gender	n (%)
Male	351 (76.64)
Females	107 (23.36)
Total	458 (100)

Medium-sized implants were among the most removed implants among the patients in 337 (73.58%) (Table 4).

**Table 4:** Types of implants removed.

Types of implants	n (%)
Small K-wires Tension band wiring Screws	99 (21.62)
Medium Intramedullary nails Plates TENS Bimalleolar implants	337 (73.58)
Large Dynamic hip screws Multiple site implant removals Spine	22 (4.80)
Total	458 (100)

The most common implant removed was related to Radius/Ulna in 132 (28.82%), and the least was related to foot 3 (0.66%) and spine 3 (0.66%) (Table 5).

**Table 5:** Anatomic locations related to removed implants.

Anatomical locations	n (%)
Clavicle	39 (8.52)
Humerus	17 (3.71)
Radius/Ulna	132 (28.82)
Femur	54 (11.79)
Patella	22 (4.80)
Tibia	98 (21.40)
Ankle	83 (18.12)
Spine	3 (0.66)
Hand	7 (1.53)
Foot	3 (0.66)
Total	458 (100)

Elective procedures were among the most common indication behind the implant removal procedures carried out in the department in 369 (80.57%) (Table 6).

**Table 6:** Indication behind the implant removal.

Indication	n (%)
Elective	369 (80.57)
Infection	30 (6.55)
Pain	18 (3.93)
Hardware prominence, soft tissue irritation	17 (3.71)
Others	24 (5.24)
Total	458 (100)

Subarachnoid Block (SAB) was a commonly used anesthetic procedure out of the total implant removal procedures in 360 (78.60%). While local anesthesia (LA) was least commonly used among 11 (2.40%). General anesthesia/Intravenous anesthesia (GA/IVA) was used on 87 (19.0%).

## DISCUSSION

Musculoskeletal disease is considered as the primary cause of physical disability globally, although the data from low and middle-income countries are not adequate. The recent figure depicts around 2.35 million people are living with musculoskeletal diseases in Nepal.^4^ There is no adequate availability of the infrastructures supporting the day-to-day and major surgical procedures in orthopedics and other departments in developing countries like Nepal. Additionally, there is also limited availability of practicing orthopedic doctors. In such a scenario, implant removal procedures occupy a significant portion of the surgical workload carried out in the orthopedic department, especially when most of those implant removal surgeries are of an elective type.

Our study depicts 17.91% of surgical procedures as implant removal procedures among all other operations in the department. A similar study conducted by Rijal et al. in a tertiary care centre of Nepal reports that 12.44% of implant removal procedures were carried out of the total orthopedic cases admitted to the hospital.^1^ Other studies also report a similar type of trend; 13.7% out of patients having prior open reduction and internal fixation operation in a study at a hospital of Ghana by Kuubiere CB et al.^3^ and 14.1% out of total major and intermediate orthopedic operations carried out three hospitals of Nigeria by Onche I, et al.^5^ Another study reports 7.83%^2^ of implant removal procedures out of total orthopedic and trauma surgery, conducted at a tertiary care center in Nepal, which is relatively low than previously discussed studies. But, in resource-limited places like Nepal, implant removal surgery tends to occupy much of the workforce in day-to-day practice.

Most of the patients, 55.68%, whose implants were removed were young adults (17-39 years) in our study. Similarly, 41.45% of patients were in the young adult group out of total implant removed patients, as reported by another study in Nepal.^2^ Another study also reports 36.1% of patients in 31-40 years age group out of total implant removed patients.^3^ Further exploration of the common age group undergoing implant removal procedures may prove helpful in the pre-allocation of the resources targeted for those age groups of people in day-to-day settings.

The study by Shrestha R et al. reports more than twice the proportion of the implant removed patients as male patients.^2^ Male patients were also twice as that of females patients whose implants were removed in other studies.^3,6^ In our study, too, there were 76.64% male patients. While 57.4% of patients were females, as reported by the study in Nigeria.^5^ This variation could occur from place to place according to the proportion of the gender in the general population. But the general trend of removing implants from male patients in a greater proportion depicts more involvement of males in physically risky activities in a major part of the globe, which reflects more orthopedic and traumatic cases among the male population.

Internal fixation with plates/screws was done in 89.4% of implant removal cases in a study.^5^ Similar trend can be observed in another study by Reith G, et al.,^7^ and Kuubiere CB.^3^ Medium type hardware, including plates and intramedullary nails, were the most common ones removed as reported by the study by Shrestha R, et al.^2^ In our study also, 73.58% of the implants removed were medium type implants. These removed implants are also among the ones that are most placed into the patients. Therefore, adequate stock of the commonly used implants will be helpful in necessary times.

Femur 27.3% followed by radius 26.9% were the commonest bone for implant removal as reported by the previous study.^2^ Other studies reported the ankle as the most common site for implant removal in 14.45%,^8^ 21%^7^ and 35.8% of the patients out of total implant removed patients.^9^ In contrast, the study by Kuubiere CB^3^ and Onche I^5^ reports 33.3% and 42.6% of the implants removed were related to femur bone, respectively. While 28.82% of the removed implants were related to Radius/Ulna and 21.40% were related to Tibia in our study. More explorative research could be conducted regarding the sites prone to frequent removal of implant regarding its optimal duration and other factors for its effective planning and counselling.

Patients' request was reported to be the most common indication for implant removal among 72.3%^5^ and 55.6%^3^ in two different studies. The patients' demand is also one of the major indications behind the implant removal in another study by Kumar K et al.^6^ While, 68% of the implant removal procedures were carried out upon the recommendation of the doctors as reported by another study.^7^ Most of the indications for removing the implants are relative and patient-driven, such as pain, prominent material, or simply request for removal as reported by one literature review.^10^ In the absence of evidence-based guidelines for the decision of implant removal, the practicing orthopedics often have to decide on their conscience and based on the availability of the limited manpower and infrastructure in daily practice.

Since the study is conducted based on the data in orthopedics department of one tertiary care center, the findings cannot be generalized to other places. A proper analytical study is also deemed necessary to explore the factors such as indications, type of implants, and anatomic locations related to implant removal to better understand the scenario.

## CONCLUSIONS

Implant removal surgery covers a major portion of orthopedic operations carried out in the department of the orthopedics of the tertiary care center under study. Most of the procedures are elective procedures done upon patients' request. In resource and manpower limited settings like Nepal, a proper long-term plan could be drafted by the corresponding hospital management and the department itself to spread out the elective surgery over the year, focusing the manpower primarily on other major operations. Proper patient counselling could also be another strategy to convince patients to postpone the medically unrequired implant removal procedures most of the time.
